# Environmental and genetic influences on early attachment

**DOI:** 10.1186/1753-2000-3-25

**Published:** 2009-09-04

**Authors:** Judit Gervai

**Affiliations:** 1 Institute of Psychology, Hungarian Academy of Sciences, 1394 Budapest, PO Box 398, Hungary

## Abstract

Attachment theory predicts and subsequent empirical research has amply demonstrated that individual variations in patterns of early attachment behaviour are primarily influenced by differences in sensitive responsiveness of caregivers. However, meta-analyses have shown that parenting behaviour accounts for about one third of the variance in attachment security or disorganisation. The exclusively environmental explanation has been challenged by results demonstrating some, albeit inconclusive, evidence of the effect of infant temperament. In this paper, after reviewing briefly the well-demonstrated familial and wider environmental influences, the evidence is reviewed for genetic and gene-environment interaction effects on developing early attachment relationships. Studies investigating the interaction of genes of monoamine neurotransmission with parenting environment in the course of early relationship development suggest that children's differential susceptibility to the rearing environment depends partly on genetic differences. In addition to the overview of environmental and genetic contributions to infant attachment, and especially to disorganised attachment relevant to mental health issues, the few existing studies of gene-attachment interaction effects on development of childhood behavioural problems are also reviewed. A short account of the most important methodological problems to be overcome in molecular genetic studies of psychological and psychiatric phenotypes is also given. Finally, animal research focusing on brain-structural aspects related to early care and the new, conceptually important direction of studying environmental programming of early development through epigenetic modification of gene functioning is examined in brief.

## Early attachment: evolutionary basis and individual variability

Early attachment, a specific personal relationship developing between an infant and the caregiver has been considered essential for survival as well as for later physical and mental development in primates including the human species [1]. The human newborn, however competent in many ways [2], cannot survive unless responsive adults feed and protect them from environmental hazards. Beyond physical care, early experiences have a significant formative influence on children's later mental health, social adjustment and personality development. Attachment theory, conceived by John Bowlby in an evolutionary framework, has proposed that the human infant is born with a set of behavioural mechanisms selected for increasing the chances of survival. The dyadic regulatory system and associated behaviours are normally activated by impending or perceived external danger and by states of internal stress, such as illness or fatigue. Thus, unlike earlier theories of parent-child relationships, which emphasized the role of (any) caregiver in satisfying the infant's physiological needs (e.g., hunger), attachment theory focuses on the selectivity of personal relationships providing protection and emotional security. The attachment behavioural system is theorised to be integrated with other behavioural systems in reaching its "set goal", the felt security of the child under various external and internal conditions. The actual behaviours of the dyadic attachment system clearly change in the course of physical and mental development of children, who come to be able to endure longer separations and increasing distances from caregivers.

Attachment relationships are formed in the course of interactions with caregivers. Infants accumulate information regarding readiness, quality and reliability of responses from others and, by the end of the first year of life, specific representations are formed about the caregivers, the self and the nature of relationships. These representations are theorised to influence children's behaviour contemporaneously, as well as influencing their subsequent social relationships [3].

### Variations of attachment patterns

Individual variations in infants' attachment to caregivers can be observed through their behaviour, especially under conditions of stress that activate seeking and maintaining proximity. The Strange Situation Procedure (SSP) [4] developed for measuring the balance between infants' exploration and attachment behaviour consists of short episodes inducing mild stress in the infant by the entrance and approach of a stranger and two subsequent brief separations from the caregiver. Dyadic behaviour videotaped throughout the session is evaluated by experts for fundamental attachment strategies [5] and for the degree of disorganization of these strategies [6]. *Secure *(B) attachment can be characterized by the infants' open communication of emotions and their ability to make use of the caregiver as a secure base from which to explore. There are two organized insecure attachment patterns; infants avoiding contact or interaction with the caregiver upon reunion and minimizing the expression of negative emotions under stress are termed *avoidantly *attached (A), whilst infants expressing intense negative emotions and wanting contact, but unable to settle following the separations are classified as *resistantly *attached (C). Infants who are unable to obtain comfort from the caregiver at times of fearful arousal, and whose behaviour in the reunion episodes appears to lack a clear strategy, may display simultaneous or sequential contradictory behaviours, misdirected, stereotypical movements, extended freezing and direct expression of fear in the presence of the caregiver. These behaviours are rated on a 9-point scale and those with a summary score > 5 are classified as *disorganized *(D).

Thousands of assessments during the last three decades have allowed the estimation of the frequencies of these infant attachment types across a range of populations. Frequencies in low social risk community samples are typically ~55% secure, ~15% avoidant, ~10% resistant and ~15% disorganized. There is some cross-cultural variation in these frequencies, but differences within any culture can be just as large as between cultures [7]. In high social risk groups exposed to severe deprivation and maltreatment [8] or among infants of adolescent mothers [9] frequencies can be dramatically different with 0-30% secure, 20-50% avoidant and resistant, and as many as 50-80% disorganized infants [8,10].

## Explanations for individual variability in attachment patterns

### Environmental factors

Bowlby's attachment theory is a truly environmental theory as it has explained individual differences in attachment patterns (attachment types) by individual variations in *caregivers' behaviour*. In their seminal study [5], Ainsworth and colleagues found links between observed care-giving behaviour at home and characteristic behaviour patterns in the laboratory-based SSP. They found that the optimal, secure behaviour pattern could be linked to sufficient sensitive responsiveness at home. Sensitivity was conceptually distinguished from responsiveness, with sensitive responses defined as being guided by an appropriate interpretation of infants' signals and changing needs. The avoidant pattern could be related to rejecting, dismissing or neglecting responses to infants' signals, especially to those signals expressing negative emotions, while in the background of the resistant pattern, unreliable, inconsistent care was identified.

Although many studies demonstrated a significant link between early care and attachment, studies varied greatly regarding in estimates of the strength of the relationship. De Wolff and van IJzendoorn [11] reviewed 66 studies to evaluate effect sizes in relation to the methodology used for assessing caregivers' sensitivity. They showed that caregiver sensitivity has been defined and operationalised in many different ways over the preceding thirty years, but however measured, it was far from being an exclusive determinant of the quality of attachment. Indeed, sensitivity accounted for less variance than was expected (effect sizes 0.24-0.32).

Over the last three decades, it has been shown that different *demographic risk factors*, especially if accumulated may effect the development of attachment, presumably through their proximal or distal influence on parenting [12]. Income and family size, parental age and education, major stressful events, such as loss of a parent, birth of a sibling, severe illness, marital relationships and breakdown affect the quality of attachment relationships [13-19].

It has been expected that secure attachment is promoted by the *psychological health *of parents, especially mothers. Empirical studies have provided contradictory results, but the majority found that mothers depressed postnatally were more likely to develop insecure attachment relationships with their infants [20-23]. Studies in general have not been able to find direct associations of mother-infant attachment with child care arrangements and with mothers' *social support *systems [12], but in high social risk groups, lack of support correlated with higher rates of insecure attachment relationships [24-26], while extensive support was found to promote security [27,28]. Security of mother-infant attachment has been found to be related to mother's *mental state *with respect to close (attachment) relationships. In several studies, the security of maternal attachment representations, as assessed by the Adult Attachment Interview (AAI) [29], has been found to be significantly related to mother-infant attachment security [30].

It has also been shown that while isolated individual risk factors may not have a significant effect on parent-child attachment, the accumulation of adversity may result in sub-optimal relationship development and insecurity of infant attachment [12]. Raikes and Thompson [31] have tested the effect of multiple social and economical factors on attachment and confirmed that their effects were mediated by mothers' care-giving behaviour. In several longitudinal studies, cumulative risk indices and life stress were used to explain discontinuities in attachment security through the life course [32-34].

Friedman and Boyle [35] have recently reviewed attachment-related findings from the longitudinal NICHD Study of Early Child Care (SECC) aiming to identify effects of timing, extent, quality, and type of child care experiences on children's development in a large sample, using well-controlled methodology. The study followed the development of more than one thousand children, of varied backgrounds, from birth and periodically assessed attachment to their mothers. Family SES and maternal sensitivity, especially in responding to the child's distress, predicted the quality of attachment at 15 months [36,37] and at 36 months of age [38] confirming the previously found moderate strength of the relationship. Recurring symptoms of maternal depression across the first three years predicted higher prevalence of insecure attachment at age 36 months [39]. Analysis of effects of non-maternal care, a special focus of the NICHD study, confirmed the lack of main effects of children's age of entry, quality of care and length of time children spent in non-maternal care, but also revealed interaction effects. The study found higher rates of insecurity if a low quality of non-maternal care was combined with low maternal sensitivity and more time spent in child care. Altogether, the conclusion of the study was that, under some circumstances, early non-maternal child care *can be *an environmental risk factor for attachment security and compromised later development.

### Environmental effects on disorganized attachment

Attachment disorganisation became a focus of developmental research when rarely occurring incoherent and contradictory infant behaviours, not fitting the Ainsworth categories, appeared to be predominant among maltreated or otherwise deprived groups of infants and young children [6,40]. Infants whose attachment strategy collapses even under the mild stress of brief separation experienced in the Strange Situation and who show high degree of incoherence and disorganisation upon reunion with their caregivers comprise on average 15% of typical populations and as high as 50-80% of high social risk groups [8]. Early disorganised attachment also proved to be one of the rare early predictors of subsequent childhood behaviour problems [41-44] and adolescent psychopathology, such as dissociative symptoms and borderline personality disorder [45,46]. Regarding the parenting background, Main and Hesse [47] proposed that caregivers of infants displaying disorganized attachment showed bouts of "frightened, threatening, and dissociative" behaviour in interactions with their infants, and this was confirmed by later studies [48-50]. In addition, Lyons-Ruth and colleagues [51] identified a broader spectrum of anomalous parental behaviour contributing to attachment disorganisation, including also communication errors, role confusion and extreme withdrawal. To some extent, these behaviours were also found in low-social-risk groups of mothers [43,52-55]. Using NICHD SECC data, Campbell and colleagues [39] have shown that chronic depression combined with low maternal sensitivity is associated with a higher prevalence of disorganised attachment in 3-year-old children. Mothers' unresolved trauma or loss has been considered as a potential source of disruptions in maternal care-giving behaviour [43,47,50,56]. According to a recent meta-analysis, however, anomalies of caregiver's mental state and behaviour had only low explanatory power in accounting for attachment disorganization [49]. Including 12 studies examining relations between maternal unresolved loss and trauma, anomalous parenting and disorganised attachment, moderate effect sizes were found for both links between maternal unresolved mental state and anomalous behaviour and infant disorganized attachment (r = .26 and .21, respectively), as well as for the link between mother's anomalous behaviour and infant disorganization (r = .34).

### Biological contribution to individual variations in infant attachment

Newborns' biologically based capabilities of self-regulation of arousal and distress states have an immediate impact on parents. The great individual variation in these capabilities can be described by dimensions such as the infant's disposition for distress or negative emotionality, irritability and soothability [57,58]. There is a long tradition of two different interpretations of the role of infant temperament in the formation of attachment relationships. Attachment theorists have suggested that temperament has no direct effect on the quality of attachment, since infant characteristics such as difficult temperament can be accommodated by sensitive caregivers, who can still foster secure attachment relationships [59]. Temperament researchers, on the other hand, have kept emphasizing that infant-caregiver interactions in the Strange Situation reflect the infant's temperament rather than the quality of the relationship [60]. In their extensive review, Vaughn and Bost [61] argue that temperament and attachment are separate constructs, and studies showing inter-relationships on the one hand, and independence on the other result from different conceptualisations and assessments of both. There is a body of empirical research results, which has demonstrated relations between attachment quality and infant irritability, proneness to distress or stress regulation [26,58,62-64]. Based on their review of literature, Mangelsdorf and Frosch [65] have suggested that effects of infant temperament on attachment may be indirect and moderated by other maternal and social variables.

Several studies have found that newborn behavioural measures are related to later secure and insecure attachment classifications [66-70]. These studies are particularly interesting because neonatal measures reflect minimal social experience. Results from the Regensburg longitudinal studies have shown that poor neonatal behavioural organisation was related to disorganised behaviour in the Strange Situation at 18 months of age [71,72]. This, together with findings that some of the behaviours characteristic of attachment disorganisation have also been found in children with developmental disorders [73,74] imply that biological factors such as the infant's capability to organise environmental stimuli, communicate and regulate internal states and behaviour may also contribute to the development of disorganised attachment. Summarizing the empirical evidence, Barnett and colleagues [75] suggested a two-dimensional model in which both biological vulnerability as well as adverse environment might contribute to the development of atypical (disorganised) attachment behaviour.

### Twin studies of attachment security and disorganisation

Since the study of parent-child attachment was so strongly driven by the environmental theory, a quantitative behaviour-genetic approach has only recently been used to investigate heritable and environmental variance components of attachment security. Most of the existing twin studies of attachment overviewed here have not been designed to focus on attachment, therefore they are quite heterogeneous regarding children's age and the method of assessment. In the Louisville Twin Study (LTS), Finkel and Matheny [76] found a significant difference between concordances of attachment classifications of 99 monozygotic (MZ) and 108 dizygotic (DZ) two-year-old twins. Model-fitting resulted in estimates of 25% genetic and 75% non-shared environmental effects. In another study of 57 MZ and 53 same-sex DZ twins aged 3.5 years, O'Connor and Croft [77] reported results of genetic model-fitting: variance estimates due to genetic, shared and non-shared environmental factors were 14%, 32% and 53%, respectively, but the genetic effect on secure vs. insecure attachment was not significant. Comparing 57 MZ and 81 DZ twin pairs, Bokhorst and colleagues [78] found only unique environmental factors accounting for the variance in disorganised vs. organised attachment, while both shared and non-shared environmental effects accounted for the variance in secure vs. insecure attachment. In a model-fitting analysis using data from 485 twin pairs, Roisman and Fraley [79] have also emphasized the role of environment (parenting quality) in accounting for the variability in toddlers' observed secure-base behaviour. Using staff/parent-rated zygosity, a model containing only shared (C) and non-shared (E) environmental variances "was able to explain the data just as well as the full [ACE] model" (p. 835) providing a heritability estimate of 0.17. Finally, using questionnaire data of attachment disorder behaviours in a very large community sample of 13,472 twins, both twin correlations and model-fitting results suggested a strong genetic influence on attachment disorder behaviour, especially in boys [80].

It is important to recognize the power constraints of quantitative genetic studies such as those using twin comparisons. These analyses aim at assessing heritability; that is, estimating how much of the population variance is due to genetic effects (the rest is environmental variance and measurement error). The twin method is based on comparison of twin correlations or concordances. If genetic effects are important for the trait in question, then correlation or concordance between monozygotic twins should be significantly greater than that between dizygotic twins. Limited sensitivity is inherent in the methodology based on detecting significant differences between twin correlations. This is not such a great limitation for many psychological and psychiatric phenotypes with substantial heritabilities of around 0.5, but may cause problems in detecting smaller, yet potentially important genetic effects. (It is estimated that any specific gene effect accounts for less than 1% of the variance of complex traits [81].) The power limitations of relatively small twin studies are not trivial. Statistical power analysis by Visscher [82] has addressed the questions of rejecting CE models within the classical twin design when the true model is ACE, i.e. there is a significant genetic variance. The simulation results have shown that necessary sample sizes become fairly large at 0.2 heritability except when shared environmental variance exceeds 0.5-0.6. Visscher and colleagues [83] have provided a web-based power calculation tool for determining the minimum number of twin pairs required to detect A and C in an ACE model of given parameters. Unfortunately, all published twin studies of attachment seem to be underpowered for detecting heritability (A), but most are powerful enough for detecting large environmental contributions.

In addition, behaviour-genetic analyses usually employ main effects models dividing up the total phenotypic variance into additive (or dominant) genetic, shared and non-shared environmental components. They normally do not separate gene-environment interactions (genetic sensitivity to environments) and gene-environment correlations (arising from experiences correlated with genetic propensities). Finally, quantitative genetic modelling does not go beyond the extent to which genetic factors influence behavioural traits, that is they are not informative about specific genes or specific environmental factors affecting the behaviours in question.

### Molecular genetic studies of attachment

The development of molecular genetic methodology started to shift the focus of study towards locating and identifying specific genes underlying genetic effects evident in twin and adoptive studies, even before sequencing the human genome was completed. The widely used strategy of allele association investigates correlations of gene variants with phenotypes, i.e., it probes if individuals with a trait of interest carry a specific gene variant more frequently than individuals in a control group (case-control studies). The method of allele association has been quickly and productively applied in the area of multi-factorial mental illnesses for which genetic components have long been demonstrated by large heritability estimates. Genes regulating the cerebral levels of important neurotransmitters (dopamine, serotonin, GABA, etc.) or signal transmission efficiency (neurotransmitter receptors and genes) have been targeted in association studies of major psychiatric disorders such as schizophrenia, bipolar disorders, attention deficit/hyperactivity disorder (ADHD), and autism [84], as well as of personality traits [85].

The first investigation of the specific genetic background of attachment behaviour showed an association between D4 dopamine receptor (DRD4) gene polymorphism and infants' attachment behaviour [86]. The level of dopamine and the density of dopamine receptors in the PFC increase between 6-12 months of infant life, when many of these functions go through intensive development [87] and when the development of first attachment relationships typically takes place. The highly polymorphic DRD4 gene has a number of frequent functional variants in the populations [88]. One of these is a variable number 48 base pair tandem repeat (48 bp VNTR) in the coding region of the gene [89]. Receptor molecules coded by the 7-repeat allele have been found to have a lower potency for dopamine-mediated coupling to adenylate cyclase than receptors encoded by the other frequent, 2- or 4-repeat forms [90], and more recent results suggest an additional effect of the 48 bp VNTR on gene expression [91,92]. Allele association studies have linked the 48 bp repeat polymorphism of the DRD4 gene with normal variations of neonatal, infant, and adult temperament [85], but also with clinical hyperactivity (ADHD) [93,94].

In the longitudinal Budapest Infant-Parent Study (BIPS), a relative over-representation of the 7-repeat variant of the 48 bp VNTR polymorphism was found in the group of infants displaying insecure-disorganised attachment behaviour with their mothers in the Strange Situation [86]. The estimated relative risk for disorganised attachment among children carrying the 7-repeat allele was four-fold, with the frequency of the 7-repeat allele being 67% in disorganised infants as opposed to 20% in securely attached infants [95], and with 50% frequencies in the insecure-avoidant and resistant groups. Subsequently, we also reported an interaction between the structural 48 bp repeat polymorphism and the -521 C/T promoter polymorphism in the same group of infants: the association between disorganised attachment and the 7-repeat allele was enhanced by the presence of the -521T allele [96]. While Dutch researchers have failed to replicate this association [97,98], parental genetic data and family-based analyses in the Hungarian sample showed a highly significant non-transmission of the 7-repeat allele (and the -521T~7-repeat haplotype) to securely attached infants, as well as a trend for preferential transmission to disorganised infants [95]. The preferential non-transmission of the 7-repeat allele to securely attached infants suggested that not carrying this allele might, in fact, have a protective effect, favouring the development of secure early attachment in this sample.

### Gene-environment interaction effects on infant attachment

Specific genetic effects on phenotypes may be conditional on specific environments and thus be undetected in other environments. Similarly, exposure to specific environments are often influenced by choices that depend on the individuals' genetic make-up. Such interplay between genes and environment may channel individual development into different trajectories early in life and affect the long-term development of mental health and disorder. A shift from association studies targeting genetic main effects towards investigating the interaction of genetic and environmental influences is reflected by the accumulating evidence for such processes being reported in recent literature [99]. Important reports are emerging, describing the moderation of behavioural responses to early rearing conditions by specific genotypes. For example, in the Dunedin study, Caspi and colleagues found that links between childhood maltreatment and later psychological maladjustment were moderated by genetic factors [100]. The functional polymorphism of the monoamine oxidase A (MAOA) gene affecting enzyme activity moderated the relation between early maltreatment and later antisocial behaviour in males. In the same population, the 5-HTTLPR regulatory polymorphism of the serotonin transporter gene was shown to moderate the effect of early maltreatment on adult depression in both sexes [101]. Both findings have since been replicated [102-104]. Importantly, in these studies, the genetic factors had no main effects on the outcome and the genetic influence was detected only when the environmental measure of maltreatment was included in the analyses.

Our own study of the genetic effects on disorganised attachment has been extended by investigating the interplay between DRD4 gene polymorphism and maternal behaviour [54]. In order to increase the range of environmental and behavioural measures, we have done this in collaboration with the Boston-based US research group led by Dr. Karlen Lyons-Ruth, an expert on disorganised attachment in infants and atypical behaviour of mothers. Demographic risk, DRD4 7-repeat genotype, levels of atypical maternal behaviour and infant disorganised attachment were combined across the middle income, low risk Hungarian and the low income, high social risk US samples (N = 96 and 42, respectively). We found that, in the combined sample, disorganised attachment was related to both cumulative demographic risk and maternal atypical behaviour, but the main effect of infant 7-repeat genotype on disorganised attachment was no longer significant. However, the relation between maternal atypical behaviour and infant disorganisation was moderated by infant DRD4 genotype. The relationship was strong in the group of infants lacking the 7-repeat variant; as expected, mothers showing a low level of anomalous behaviour had infants displaying a low level of disorganisation, and conversely, infants of highly atypical mothers showed a high level of disorganised attachment behaviour. In contrast, the level of disorganisation in infants who carried the 7-repeat allele was at an intermediate level and unrelated to the degree of maternal atypical behaviour. Infants carrying the 7-repeat allele thus seemed to be less sensitive to maternal behaviour. At the same time, the previously reported main effect of the 7-repeat genotype on attachment disorganisation was restricted to the group with mothers low on atypical behaviour. This pattern of results was also present in the separate US and the Hungarian samples. We hypothesised that functional variations in the DRD4 gene expressed preferentially in brain regions of the reward circuit might modulate sensitivity to maternal stimuli, which in turn might result in infants' differential sensitivity to aspects of care-giving behaviour.

Results of this first molecular genetic study of infant attachment [86,96] seem to have transformed the attachment field by increasing interest in studying genetic and gene-environment interaction effects. A number of research groups have genotyped participants of previous and ongoing attachment studies, or have begun including genetic markers in the design of new studies. A further investigation of the DRD4 48 bp VNTR showed that infant genotype moderated the previously reported intergenerational transmission of mothers' unresolved trauma to disorganised attachment: the link was significant for infants carrying the 7-repeat allele only [105].

Studies have been extended by investigating associations with other candidate genes. The polymorphic serotonin transporter gene, coding for one of the important regulator of the synaptic level of the neurotransmitter serotonin, has been linked previously to anxiety-related traits [106] and affective disorders [107]. The 5-HTTLPR repeat polymorphism in the promoter region of the gene affects gene expression, that is the number of available serotonin transporter molecules. The 'short' 5-HTTLPR allele has been associated with low serotonin metabolism and behavioural problems in infant and juvenile Rhesus monkeys reared with peers only, but not in monkeys who were reared with their mothers and peers during infancy. In contrast, monkeys carrying only the 'long' allele showed normal metabolism and behavioural functioning, regardless of their early rearing history [108]. A similar protective effect of the homozygous 'long/long' (l/l) genotype or else a buffering effect of maternal behaviour was found in human studies of infant attachment. For infants with a 'short' allele, variation in mothers' responsiveness was significantly associated with attachment security [109] or attachment disorganisation [110,111]. For infants homozygous for the 'long' allele (l/l), there was no association between maternal responsiveness and attachment security or disorganisation.

The gene-environment interaction effects on attachment reported in the above-cited publications are consistent with Belsky's differential susceptibility hypothesis [112], i.e., children's susceptibility to care-giving experience seems to be moderated by genetic factors. Unfortunately, all these attachment studies, as usual, involved relatively small sample sizes. In the light of the latest meta-analyses questioning the 5-HTTLPR main effects [113] and 5-HTTLPR by stressful environment interaction effects [114], these initial investigations of genetic and gene-environment interaction effects on attachment need to be confirmed by larger studies, possibly including multiple polymorphisms of multiple candidate genes.

## Genetic moderation of effects of early mother-child relationship on children's problem behaviour

Following up their above mentioned study [109], Kochanska and colleagues [115] found that the 5-HTTLPR promoter genotype moderated not only the relationship between maternal responsiveness and attachment security to the mother, but also the relationship of attachment security with children's later ability for self-regulation at 2, 3 and 4 years of age. Insecurely attached children with a 'short' 5-HTTLPR allele (s/s and s/l) developed poor regulatory capacities, but those who were securely attached did not show a deficit compared to children who were homozygous for the 'long' allele (l/l). For children homozygous for the 'long' 5-HTTLPR allele, attachment security was not related to self-regulation. The authors' interpretation that secure attachment relationship can serve as a protective factor in the presence of risk conferred by a genotype might however be confounded by their previously reported finding [109] that infants with l/l genotype were predominantly securely attached. It seems that in this case, consistent with other primate and human studies, the 5-HTTLPR l/l genotype has a protective effect in the face of parenting risk.

In a small-scale study, (N = 47), infants' DRD4 genotype was found to moderate the relationship between maternal insensitivity and externalising child behaviour: children who carried the 7-repeat allele and had relatively insensitive mothers showed the highest level of externalising behaviour [116]. In a further study, the same researchers investigated the effect of children's DRD4 genotype on the efficacy of parenting intervention in a pre-selected 'externalising' sample [117]. At the follow-up stage, they found that intervention aimed at increasing parental sensitivity and positive discipline was effective in reducing externalisation for children with a 7-repeat allele, but not for children without the 7-repeat allele. This findings points to a genetically moderated differential susceptibility to intervention, which might partially explain difficult-to-treat cases often encountered in clinical work.

## Methodological problems in molecular genetic studies

Genetic association studies in the field of psychiatric and psychological genetics have been suffering from inconsistencies in replications of results. There can be many reasons for these difficulties, some of which are the result of methodological flaws, for example genotyping errors. Improved measurement of phenotypes and carefully controlled genotype determination can reduce the danger of false negative findings [118]. Failure of replication, however, may have 'legitimate' underlying causes. Initially, replication problems of case-control studies were often attributed to population stratification (case and control groups originating from genetically distinct populations) and to multiple testing without appropriate corrections, which could result in spurious associations. These problems have been largely dealt with by using genetic data from families [119] and more stringent statistical testing [120]. However, the real problem might be that more complex behavioural phenotypes are affected by many genes, each with only a small effect accounting for only 1% or even less of the phenotypic variance [81], therefore inadequate statistical power is a serious problem. Likewise, different environment interactions across samples can also hinder replication of an association. A considerable genetic heterogeneity is expected within and among studies, as different combinations of the various alleles of the multiple genes and epistatic gene interactions may produce similar phenotypes. This heterogeneity may go undetected as often in smaller studies only a few genetic markers are investigated. According to Greene and colleagues [121], real associations may not replicate in independent samples if they are part of a larger epistatic interaction. In these cases, small sample differences in allele frequency at an interacting locus may impact the power negatively, so the originally reported effect may not replicate or even reversed. The recommendation is to check for interaction with other polymorphisms (see, for example the replicability of 'novelty seeking', where interaction of at least three gene loci made replication uncertain [122].

As mentioned above, some of these problems can be tackled by increasing study sizes and combining study samples, increasing the number of carefully chosen candidate polymorphisms or as Plomin and Davis suggest [81] employing the newly available genome-wide association (GWA) strategy. In GWA studies many hundred thousand single nucleotide polymorphisms (SNPs) across the genome of many (often more than 1000) individuals are genotyped. Comparing profiles of 'case' and 'control' groups, SNPs associated with caseness can be identified. GWA studies of type II diabetes identified nearly 20, robustly replicating gene loci. The associated alleles were common in the studied populations and all had only small effects on disease risk [123]. Although, this seems to be the "future of genetics in psychology and psychiatry" [81], GWA is almost certainly unfeasible in the field of attachment studies.

## Animal research pointing to new directions: environmental modification of brain anatomy and gene activity, epigenetic effects of parenting

Studies in animal models have found convincing evidence for the critical impact of early emotional experiences. Studies from the 1950s showed that even short separations of young rodent pups from their mother have profound and persistent effects on behaviour and physiological stress reactivity [124]. In the last two decades, brain development shaped by the interplay of genetic predispositions and experience-induced adaptation has been extensively studied primarily in the context of stress elicited by early separation from the primary caregiver.

An avian model of early parent-offspring bonding is filial imprinting in precocious birds which is accompanied by extensive reorganization in the frontal lobe. Domestic chicks imprinted on artificial stimuli in experimental settings showed increased synaptic connectivity in the intermediate medial hyperstriatum ventrale (IMHV), which seems to be important for storage of memory acquired during imprinting [125,126]. Bock and Braun [127] showed that successful imprinting in chick is accompanied by extensive pruning of excitatory spine synapses in other associative forebrain regions. Imprinted animals later responded to the presentation of the learned stimulus with enhanced brain electrical and metabolic activity (see Sullivan et al. [128] for a review).

This experience-dependent development of neural connections serves the adaptive response of the offspring to its actual normative environment. However, in case of adverse early influences, the same plasticity may lead to altered neural development with long-lasting behavioural and physiological effects. There has been accumulating evidence for the importance of parental care, especially tactile stimulation in the subsequent development of infants of mammalian species. Separation from the mother in rodents induces physiological and behavioural responses including vocalisation and searching behaviour, corticosterone hormone release and inhibition of metabolism related to growth and later stress reactivity [129,130]. Bock and colleagues [131] found synaptic changes in the prefrontal cortex of rat pups exposed to 1-hour-long separations from their mother. These changes were region-specific, depended on timing of the separations during the first weeks of the pups' life, and they also found a possible link between synaptic changes and endocrine function. A further study on the semi-precocious rodent Octodon degus [132] showed that short, repeated separations in the first three weeks of life resulted in significant alterations of density of neurons releasing corticotropin releasing factor (CRF) in brain regions involved in emotion regulation. More sustained early social isolation of young degus altered the serotonergic and dopaminergic cortical innervation in the orbital prefrontal cortex possibly reflecting different functioning of these monoamine transmitter systems in result of parental deprivation [133].

These few examples selected from an extensive research field illustrates the critical effects of early stress on brain development. The mechanisms underlying alterations in brain development are far from being understood in animals and even less in humans, but new molecular techniques and imaging methods may hold the key to growing knowledge of normal and corrupted developmental trajectories.

In relation to the association of infant attachment with a promoter polymorphism of the serotonin transporter gene, we have mentioned that gene expression may be affected by variation in the DNA sequence of the regulatory region of the gene. There is, however, increasing evidence for changes in gene expression through DNA and chromatin modifications *without *a change in the inherited nucleotide sequence. Epigenetic modifications of promoter regions that influence the transcription of genes coding for crucial protein products can be induced by the environment. Although the mechanisms through which these modifications occur have yet to be fully explored, one important process is environmentally induced DNA methylation leading to transcriptional silencing. It has also been shown that these environmentally mediated effects on gene expression tend to persist in the individual's lifetime and can even be transmitted from one generation to the next [134]. The pioneering research by Meaney and colleagues [135] has shown that, in rats, stable individual differences in maternal care are related to individual differences in pups' later behaviour and stress reactivity. Cross-fostering experiments have proved that these maternal effects are not heritable in the narrow sense, but are consequences of the characteristics of maternal care received in the first week of life. Meaney and colleagues have found that variation in early care affects the expression of the glucocorticoid receptor gene in the hippocampus by differential methylation of the promoter region of the gene leading to stable differences in offspring stress reactivity persisting through adulthood [136]. Moreover, they have shown that, through differential patterns of DNA methylation of the promoter region and thereby differential expression of the estrogen receptor alpha gene in the medial preoptic area (MPOA) of the brain, this individual variation of early experience influences adult reproductive behaviour, suggesting a mechanism for intergenerational transmission of the pattern of maternal care [137,138]. Results of animal work cannot be translated directly to humans, but initial findings of recent research suggest an effect of parental care on epigenetic regulation in the human brain. The methylation pattern of the promoter of glucocorticoid receptor gene in the hippocampus of suicide victims with a history of childhood abuse has been found to differ from that of suicide victims with no childhood abuse or control subjects [139]. Thus, there is a good reason for hypothesising that epigenetic modification of gene expression plays a role in the development of early mother-infant relationship, even if at present it seems impossible to study these processes in human infants.

## Conclusion

There is a considerable individual variation in infants' attachment behaviour with their primary caregivers. Attachment theory, as first conceived, gave an environmental explanation for this variation, regarding caregivers' sensitive responsiveness to infant signals as the crucial factor for the development of optimal, secure attachment. This exclusively environmental explanation has recently been challenged by studies that have included physiological and genetic measures. Although twin studies of attachment are providing mixed results, molecular genetic studies are showing that specific genetic polymorphisms of the dopaminergic and serotonergic neurotransmisson systems may moderate the statistically identified links between caregivers' behaviour and infant attachment. These gene-environment interaction effects, pointing to genetically-based differential susceptibilities to care-giving environments, have been more often found for disorganised attachment, which is an extreme form of insecure attachment predictive of subsequent behavioural problems and psychopathology. Because the results of the as-yet few and relatively small-scale studies are not wholly consistent, there is a clear need for larger-scale and carefully designed studies that examine multiple polymorphisms of multiple candidate genes. More recent research on the epigenetic modification of gene expression by early maternal care in animals suggests the possibility of similar processes affecting human development. The investigation of such processes in humans, although not feasible at present, would offer an opportunity to gain a deeper understanding of developmental psychopathology and the intergenerational transmission of attachment and parenting.

## Competing interests

The author declares that they have no competing interests.
